# Role and Applications of Experimental Animal Models of Fontan Circulation

**DOI:** 10.3390/jcm13092601

**Published:** 2024-04-29

**Authors:** Zakaria Jalal, Elise Langouet, Nabil Dib, Soazig Le-Quellenec, Mansour Mostefa-Kara, Amandine Martin, François Roubertie, Jean-Benoît Thambo

**Affiliations:** 1 Department of Pediatric and Adult Congenital Cardiology, University Hospital of Bordeaux, 33600 Pessac, France; nabil.dib@chu-bordeaux.fr (N.D.); francois.roubertie@chu-bordeaux.fr (F.R.); jean-benoit.thambo@chu-bordeaux.fr (J.-B.T.); 2 LIRYC Electrophysiology and Heart Modeling Institute, Fondation Bordeaux Université, 33600 Pessac, France; 3 Centre de Recherche Cardio-Thoracique de Bordeaux, INSERM U1045, 33600 Pessac, France; 4 Department of Pediatric and Adult Congenital Cardiology Anesthesiology, University Hospital of Bordeaux, 33600 Pessac, France; elise.langouet@chu-bordeaux.fr; 5 Centre Cardio de Bretagne Occi, 29200 Brest, France; soaz-lq@hotmail.fr; 6 Adult Congenital Heart Disease Medico-Surgical Unit, European Georges Pompidou Hospital, 75015 Paris, France; mansourmostefakara@gmail.com; 7 Department of Cardiac Surgery, University Hospital, 97400 Saint-Denis, France; amandine.martin@chu-bordeaux.fr

**Keywords:** univentricular heart, Fontan procedure, animal model, experimental model

## Abstract

Over the last four decades, the Fontan operation has been the treatment of choice for children born with complex congenital heart diseases and a single-ventricle physiology. However, therapeutic options remain limited and despite ongoing improvements in initial surgical repair, patients still experience a multiplicity of cardiovascular complications. The causes for cardiovascular failure are multifactorial and include systemic ventricular dysfunction, pulmonary vascular resistance, atrioventricular valve regurgitation, arrhythmia, development of collaterals, protein-losing enteropathy, hepatic dysfunction, and plastic bronchitis, among others. The mechanisms leading to these late complications remain to be fully elucidated. Experimental animal models have been developed as preclinical steps that enable a better understanding of the underlying pathophysiology. They furthermore play a key role in the evaluation of the efficacy and safety of new medical devices prior to their use in human clinical studies. However, these experimental models have several limitations. In this review, we aim to provide an overview of the evolution and progress of the various types of experimental animal models used in the Fontan procedure published to date in the literature. A special focus is placed on experimental studies performed on animal models of the Fontan procedure with or without mechanical circulatory support as well as a description of their impact in the evolution of the Fontan design. We also highlight the contribution of animal models to our understanding of the pathophysiology and assess forthcoming developments that may improve the contribution of animal models for the testing of new therapeutic solutions.

## 1. Introduction

The Fontan procedure, also known as total cavopulmonary connection, was first published in 1971 by Francis Fontan and Eugène Baudet [1]. Initially developed to palliate tricuspid atresia, the technique is now applied to a wide range of congenital heart diseases in which two-ventricle circulation cannot be achieved. The Fontan circulation consists of bypassing the right atrioventricular system by connecting the inferior vena cava (IVC) and superior vena cava (SVC) to the pulmonary arteries (PAs) such that the venous return and transpulmonary circulation flow passively owing to high central venous pressure, low pulmonary vascular resistance, and good systolic and diastolic systemic single-ventricle (SV) function [2]. While the procedure has allowed these patients to survive and reach adulthood in more than 40% of cases, along with a 30-year survival rate of approximately 85% after Fontan surgery as of 2020 [3], it still presents complications due to the pathophysiology of this palliation. In the short term, perioperative complications reach 26% with 4% mortality [4], while in the long term, patients are likely to develop congestive liver failure, pulmonary vascular remodeling due to non-pulsatile blood flow, kidney failure from cardio-renal syndrome, plastic bronchiolitis, and coagulation [5,6]. Once these complications become global and irreversible, they fall under the umbrella term “failing Fontan” [5,7]. The only curative treatment option is a heart transplant. Despite it being 50 years since the invention of this procedure, research has failed to propose an effective and durable solution for these patients.

Among different research methods, the animal model has been essential in supporting us to fully understand the Fontan physiology and its complications, as in the cases of the Tetralogy of Fallot [8,9] or the atrial septal defect [10,11].

This review aims to present a comprehensive overview of the various animal models used in Fontan procedural research according to their objectives, the surgical models used and their technical improvement, understanding of the mechanisms of failing Fontan from hemodynamic, pulmonary endothelium, and electrophysiological perspectives, and the models that allow for the development of new therapeutic and assistance strategies.

## 2. Methods

A systematic literature search for relevant full-text publications in English was conducted in the electronic PubMed database on 10 February 2023. The following search terms were used: “Fontan animal model”, “TCPC animal model”, “Fontan experimental model”, “total cavopulmonary connection animal model”, “total cavopulmonary connection experimental model”, and “TCPC experimental model”. The publication period was limited to between 1 January 1990 and 10 February 2023.

This review aimed to identify all studies reporting right heart bypasses in animal models. To select appropriate studies, the inclusion criteria were defined as an experimental accomplishment of right heart bypass (atrium, ventricle, or both) in a large acute or chronic animal model reported in a full-text paper in English; and the exclusion criteria were clinical trials, in vitro and in silico studies, and non-English-language articles.

In this review article, we present an overview of Fontan animal models according to their objectives, to better highlight their positive contribution but also to discuss their potential limitations. For that reason, it is logical to focus on animal studies and to exclude human and in vitro/in silico experiments.

In a first step, three investigators independently screened the titles and abstracts of all studies. Each study was considered eligible if it matched the inclusion criteria. Table A1 displays the main features of the most relevant studies included in this review.

## 3. Animal Models Involved in the Technical Evolution of the Fontan Procedure

**Figure 1:**
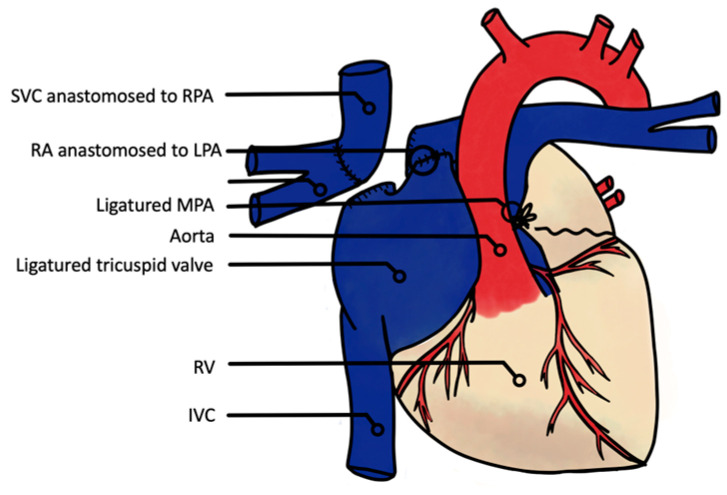
First-in-human surgical atriopulmonary connection by Fontan et al. SVC derivated to the RPA, and RA to the LPA, using the RA as a pump [1]. SVC: superior vena cava; RA; right atrium; RV: right ventricle; IVC: inferior vena cava.

**Figure 2:**
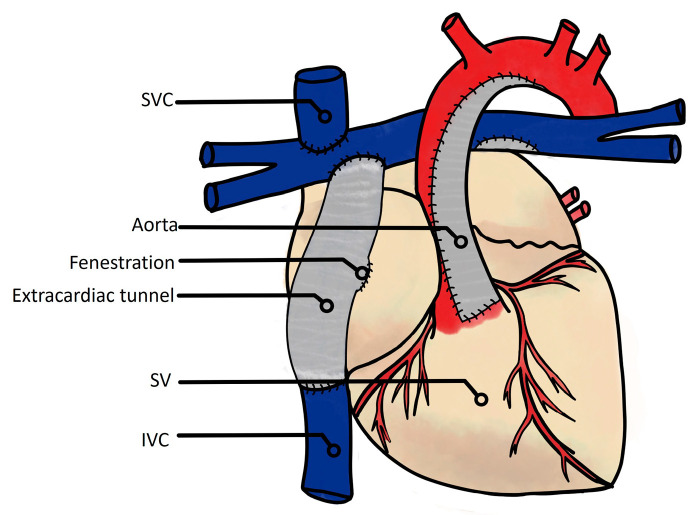
Current total cavopulmonary connection with extracardiac tunnel. SVC: superior vena cava; SV: single ventricle; IVC: inferior vena cava.

From the first description of Fontan and Baudet in 1971, many modifications to the Fontan operation have been reported with different conduit positions and materials. Nowadays, this surgical program consists of a total cavopulmonary connection (TCPC), with lateral tunnel or extracardiac conduits (Figure 1 and Figure 2).

The first animal model was conceived by Haller et al. in 1966 in dogs [12], in which the SVC and right pulmonary artery (RPA) were anastomosed, the tricuspid valve closed, and/or the right atrium (RA) anastomosed to the main pulmonary artery (MPA), similarly to Fontan et al., who described the first atriopulmonary connection (Figure 1) [1]. The question of the need for a valved conduct between the RA and LPA to avoid regurgitation and optimize the pump effect of the atria was studied by Shemin et al. in 1979 [13], who compared this montage with valved or non-valved RA-LPA conducts and concluded that the main factor influencing conduit flow was right atrial pressure, without a significant effect of valve implantation. This was likely due to the absence of valve closing, which is in line with clinical studies [6]. This pioneering montage given way in clinical use to a “pumpless” one, replacing the atrio-pulmonary connection by connecting the IVC and SVC to the pulmonary arteries.

One remaining question about the TCPC and extra-cardiac conduct is the timing between setting up the bidirectional Glenn procedure (BDG) and the TCPC. In 2001, Tanoue et al. noted that patients who went through a Glenn shunt (SVC to the RPA) procedure prior to the TCPC seemingly had better outcomes. The authors hence designed a protocol to test the benefit of performing a bidirectional Glenn procedure (BDG) preceding the TCPC and compared the contractility and mechanical efficiency of the SV between two-staged palliation and a primary TCPC. According to their preclinical study results, arterial–ventricular coupling measured by the Ea/Ees ratio (Ea: arterial elastance representing the afterload, and Ees: representing ventricular contractility) improved in the two-stage group [14]. The resulting outcomes concurred with the clinical data and showed better mechanical effectiveness in the two-stage group, in line with clinical results of the staged Fontan procedure in humans [15].

**Figure 3:**
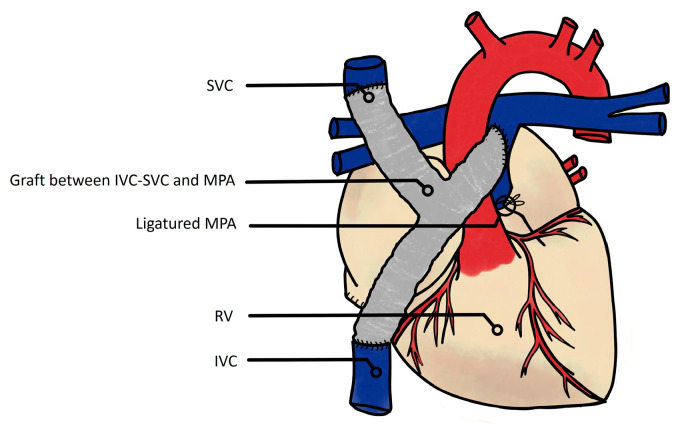
Kanakis et al.’s surgical model of the Fontan circulation without cardiopulmonary bypass, showing Y-shaped conduct between both the vena cava and MPA [16]. SVC: superior vena cava; MPA: main pulmonary artery; RV: right ventricle; IVC: inferior vena cava.

An additional challenge in the improvement of the surgical technique was avoiding the use of a cardiopulmonary bypass, achieved via two different strategies. The first strategy, consisting of a TCPC without cardiopulmonary bypass (CPB), was tested in 2011 by Kanakis et al. in eight pigs using a Y-shaped conduit anastomosed with the IVC, SVC, and MPA (Figure 3) [16]. Inotropic support was required during the procedure and withdrawn within less than one hour after completion; however, significant hemodynamic changes were observed once the TCPC was completed, with pulmonary vascular resistance multiplied by nearly twofold, and cardiac output down to about 50%. This study showed the feasibility of off-pump TCPC, despite being technically demanding, while warranting greater emphases on hemodynamics and surgical techniques [16].

**Figure 4:**
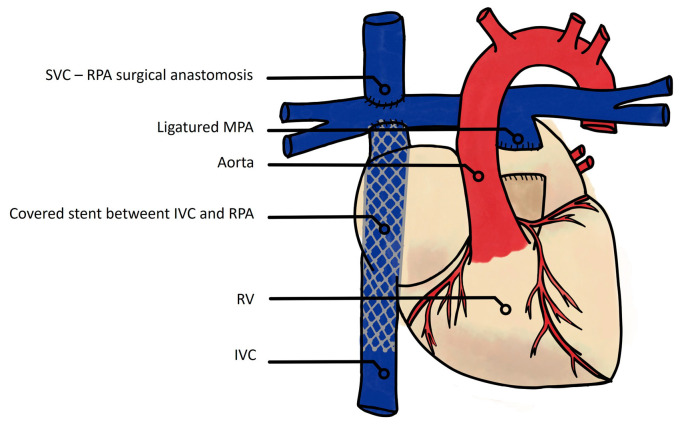
Klima et al.’s hybrid model of the Fontan circulation without cardiopulmonary bypass. First step: surgical reimplantation of the SVC in the RPA, and ligature of the MPA; second step: transcatheter implantation of a covered stent between the IVC and RPA [17]. SVC: superior vena cava; RPA: right pulmonary artery; MPA: main pulmonary artery; RV: right ventricle; IVC: inferior vena cava.

Alternatively, some teams aimed to avoid CPB by achieving a Fontan circulation using interventional catheterization techniques. The first attempt was described by Klima et al. in 10 sheep and consisted of a two-step hybrid procedure. The first surgical step comprised of a “pumpless” anastomosis between the SVC and RA to the RPA and a ligature of the MPA. The second step was performed on the same day with a transcatheter implantation of an intracardiac covered stent between the IVC and the RA-RPA anastomosis (Figure 4) [17]. All animals survived the hybrid procedure, although no hemodynamic studies or wake-up attempts were performed. The issue of coronary drainage by the coronary sinus precluded by the ligature of the RV-PA outflow tract was not explored [17]. Subsequently, in 2005, Konstantinov et al. also described a hybrid technique, with the surgical step requiring both CBP and the same anastomosis of the SVC and RA to the RPA but with preservation of the RV-PA connection, followed by similar transcatheter completion, i.e., covered stent implantation between the IVC and SVC. All animals survived the procedure, but without a wake-up attempt or hemodynamic observations [18]. These hybrid models result in very similar Fontan circulations to the modern TCPC on the anatomical and hemodynamic sides.

Between 2011 and 2014, a team from the Necker Hospital led by Y. Boudjemline developed a new hybrid procedure in sheep with dedicated devices and without the need for CPB [19]. By first using the same strategy with direct SVC-RPA anastomosis and a specially designed occluded stent between the RPA and the RA, subsequently completed on the same day with a covered stent deployed between the IVC and the SVC through the occluded stent, the authors proved the feasibility of this technique as an acute and unawakened animal model [19]. After (unpublished) failures of several strategies of a staged hybrid technique of an SVC to RPA surgical connection (conduit between the SVC and RPA and occlusion of the SVC-RA junction; Y-conduit between the IVC-SVC and RPA), the authors focused on the improvement of the transcatheter completion by creating a modified Glenn model with a “safety SVC-RA conduit”, which they used to perform and test several transcatheter completions and assess the latter at an early stage and in the long term, after various healing periods [20]. In 2013 and 2014, the same group used this model and reported a comparison of two different extracardiac completions after one to three months [21]. These extracardiac hybrid models were comprised of (1) one group with a sutured Gore-Tex tube between the SVC and the IVC filled with heparinized saline, with completion by transcatheter stenting of the Gore-Tex–caval junctions and occlusions of native atrio–caval junctions, and (2) a second group in which the tube was fenestrated in the RA, allowing blood flow prior to completion using a covered stent to clog the fenestration. Some of the animals were sacrificed immediately after completion, and the remaining animals after three months. The authors observed a complete occlusion of the Gore-Tex conduit by fibrotic tissues in the non-circulant group, and there was 100% survival for the circulant extracardiac tube; after completion, circulant extracardiac tube animals were followed until three months and showed no complications except an occlusion of the “safety SVC-RA” conduit by pectinate muscle without any impact on extracardiac TCPC functioning [21,22].

In a further study involving a transcatheter Glenn shunt using magnetic catheters, a team from UCLA succeeded in opening a covered stent between the SVC and RPA in two of three swine, although one of the two swine died from pericardial effusion [23]. In Germany, in 2011, Schmitt et al. published the results of six transcatheter Glenn completions using traditional angiography catheters, with satisfying results for four of the completions without incident at the end of the procedure. Despite the poor outcomes of this model, the notion of achieving a percutaneous Glenn shunt in association with TCPC transcatheter completion without CPB or open chest surgery represented a huge step forward in the innovation of univentricular palliation and a future direction [24].

Another innovative approach to the Fontan circulation procedure tested in animals was cardiomyoplasty. Morita et al., in 2001 [25], designed a “modified-Fontan” surgical model consisting of an initial 6-week electrical preconditioning of the left latissimus dorsi in dogs, after which surgical RV obliteration, tricuspid valve closure, and RPA ligation were performed. The RA and RPA were then anastomosed with an aortic homograft incorporated with a pericardial pouch as a compression chamber fixed on the RV epicardial surface. After termination of the bypass, the latissimus dorsi was applied to wrap the pericardial pouch and ventricle clockwise and was stimulated at a 1:1 ratio with the heart rate [25]. Hemodynamic data were collected for 4 h and showed an improved pulmonary output under stimulation. In 2002, Voss et al. conceived a similar model in foxhounds, except for the use of a valveless tube between the RA and the RPA. Their results were overall similar, with an improvement in PA flow associated with an increase in central venous pressure (CVP) [26]. These preclinical studies using cardiomyoplasty, although still necessitating highly invasive procedures (open chest surgery and CBP), nonetheless provided new perspectives on the improvement of the Fontan circulation procedure.

## 4. Animal Models Assessing Fontan-Circulation-Associated Complications

### 4.1. Hemodynamic Features

**Figure 5:**
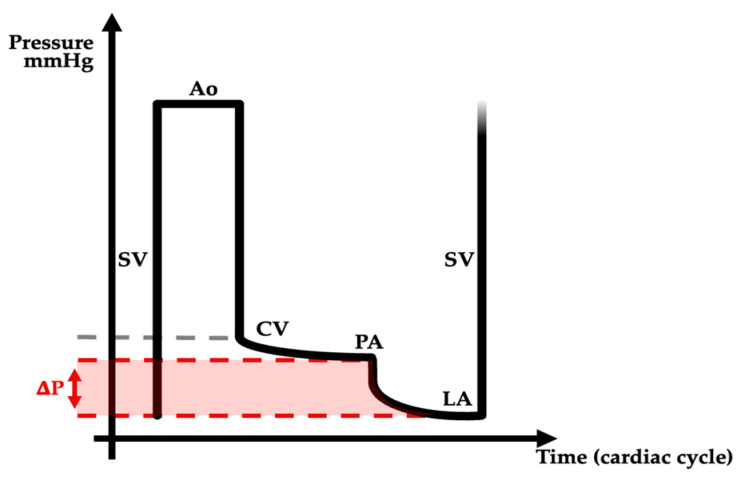
Main Fontan circulation hemodynamic features displayed as a pressure/time diagram over a cardiac cycle. SV: single ventricle, Ao: aorta, CV: caval veins, PA: pulmonary artery, LA: left atria, ∆P: transpulmonary gradient.

In Fontan patients, the single ventricle provides unique power to allow blood flow to circulate from caval veins through the pulmonary circulation towards the left atria and finally the aorta (Figure 5). Several works on animal models studied a variation in one these parameters in order to better understand the Fontan physiology.

The first published animal model of the Fontan circulation designed to understand this singular hemodynamic physiology was achieved by Kaku et al. in 1990. This team designed a Fontan dog model without the need for CPB, with an end-to-side anastomosis between the SVC and RPA and an extracardiac connection from the IVC to the SVC, with coronary sinus blood diverted to the left ventricle using a silicone conduit [27]. Once the model was surgically achieved, the authors measured systemic arterial pressure, mean pulmonary arterial pressure, cardiac output, central venous pressure, and coronary blood flow in order to calculate systemic vascular resistance (SVR) and pulmonary vascular resistance (PVR) under four central pressure conditions (regulated by fluid loading), and subsequently under norepinephrine (alpha agonist), isoproterenol (beta agonist), and phentolamine (alpha antagonist). Their findings highlighted the preload dependency of the Fontan circulation, as witnessed by a higher cardiac output and lower pulmonary vascular resistance in the case of high central venous pressure, although a threshold effect was not determined. An increase in cardiac output in peripheral and pulmonary vasodilatation as well as a proportional effect of norepinephrine on pulmonary vascular resistance were also observed. A noteworthy finding was the need for abdominal contention in their animals to counteract splanchnic vasodilatation and allow for achieving an increase in central venous pressure [27].

In 1993, Haneda et al. also explored the hemodynamic changes after Fontan surgery (in mongrel dogs), with and without the presence of the right heart, by placing a suture string tourniquet around the tricuspid valve and excluding the right ventricle by tightening the suture [28]. The authors reported a drop in cardiac output and a significant increase in PVR and left atrial pressure (LAP) during the right ventricle bypass; no intervention such as fluid loading or vasoactive drugs was administered during this study. The main criticism of this model is the preservation of the atrium and atrial systole, thereby allowing minimal pulsatility and facilitating transpulmonary flow, which they described as a “prominent ‘a’ wave” [28]. In 1995, Macé et al. aimed to study the hemodynamics of three different degrees of right heart bypass in a dog model. Their surgical model consisted of an end-to-side anastomosis with a Y-tube between the SVC-IVC and MPA with accompanying snares around the SVC and IVC to successively change the bypass; a large atrial septal defect was hence created and a silicone conduit was added between the RV and the LA to drain the remaining blood flow [29]. Their findings underscored a significant decrease in arterial pressure and cardiac output between the superior cavopulmonary shunt, inferior cavopulmonary shunt, and bicavopulmonary shunt, with a low left atrial pressure and a significant increase in pulmonary artery pressure. A significant increase in the left atrial pressure through fluid loading was required to maintain a stable cardiac output at about 2 L/min/m^2^, which confirmed that the left ventricular inotropic function was preserved [29]. In 2000, Macé et al. used another TCPC design in pigs with a similar Y-tube between the SVC, IVC, and MPA and an adaptable mitral valve ligature, which allowed for collecting data under various left atrial pressure conditions [30]. The authors notably explored the relationship between central venous pressure, pulmonary arterial pressure, left atrial pressure, venous return resistance, and cardiac output, and determined the mean circulatory filling pressure. They observed a significant increase in pulmonary vascular resistance and pulmonary arterial pressure with the Fontan circulation compared to controls (biventricular hearts) and thus concluded on the importance of the gradient between the mean circulatory filling pressure and pulmonary arterial pressure in the Fontan circulation [30]. Ketner et al. studied the cardiopulmonary interaction in lambs using a procedure most similar to the current TCPC, performed under CPB, in order to integrate the results into a computational model. They collected MPA, superior vena cava pressure, inferior vena cava pressure, right atrial pressure, and left atrial and left ventricular pressures by measuring the cardiac output and concluded that the energy loss in the TCPC was most important for positive-pressure ventilation at a high frequency and low tidal volume [31].

In 1997, Lardo et al. explored the hemodynamic effect of right atrial dilatation in the TCPC on an explanted sheep heart model with an atrio-pulmonary connection method (connection between right atrium and main pulmonary artery with tricuspid valve closure) [32]. Using an extracorporeal circulation to control the right atrial pressure and dilatation, and by measuring the pressure inside the superior and inferior vena cava and right pulmonary artery, the authors demonstrated an increase in the rate of energy (fluid) losses in parallel with an increase in right atrial volume, which could represent one of the mechanisms of the failing Fontan in atrio-pulmonary connections [32].

In 2002, Szabo et al. published a study investigating changes in preload, afterload, and contractility effects in the Fontan procedure. Using a canine model with a Y-tube from the SVC-IVC to the MPA, they measured end-systolic elastance and stroke work and highlighted an increase in the ventriculoarterial coupling ratio, which translated into reduced mechanical efficiency following the Fontan procedure and a narrow margin of ventricular adaptation to hemodynamic changes [33].

### 4.2. Pulmonary Vascular Dysfunction

In order to evaluate the underlying mechanisms of Fontan-associated pulmonary vascular disease, Malhotra et al. assessed the expression of pulmonary angiotensin-converting enzyme (ACE) after a unidirectional cavopulmonary anastomosis between the SVC and RPA in a lamb model [34]. They reported a significant decrease in the level, expression, and activity of ACE during the first 15 weeks of follow-up, and a subsequent normalization of ACE afterwards. In an ensuing publication, the authors examined the level of angiotensin II (AT-II) receptors in the same model. They found a rapid elevation of AT-II receptors in the lung concomitant with a high density of these receptors on the pulmonary artery endothelium. Intrapulmonary shunts on contrasted ultrasound echocardiography were also found, which could be explained by the aforementioned changes in angiotensin levels [34,35]. Another team led by Y. Zongtao studied factors linked to endothelial dysfunction and vascular remodeling using a canine unidirectional cavopulmonary shunt model with end-to-end anastomosis between the SVC and RPA and comparing the right and left lungs as a control after three months [36]. Histological examination revealed a significantly higher expression of endothelin-1 and nitric oxide and structural modifications including an increased thickness of the vascular wall in the right lung compared to the left lung. These differences were putatively explained by the loss of pulsatility. In a second study, the authors performed a histological analysis six months after the cavopulmonary connection with the same observations on nitric oxide production and vascular remodeling [36]. With regard to the endothelial complication of the TCPC, Henaine et al. studied the semi-quantitative effect of the loss of pulsatility three months after surgery, by comparing three groups of pigs: (i) a sham group, (ii) a group with a unidirectional cavopulmonary shunt between the SVC and RPA (nonpulsatile), and (iii) a group with a bidirectional cavopulmonary shunt between the SVC and PA and a preserved RV outflow (pulsatile). The initial results showed gradual and significant effects on the PVR and PA pressure and histological effects on arterial wall thickness and arteriovenous malformation, which appeared proportional to the loss of pulsatility [37].

### 4.3. Electrophysiological Studies in Fontan Circulation

The Fontan circulation is associated with a high risk of rhythmic complications, particularly supraventricular tachycardia. In addition, the repercussion of losing atrial contractility is Fontan-circulation-related hemodynamic failure, leading to an increased central venous pressure and failing Fontan circulation.

A team from Washington studied this issue and published a series of articles using a canine model of intracardiac lateral tunnel Fontan circulation, also called the modified Fontan, with right atrial sutures simulating the construction of the lateral tunnel [38,39]. When needed, they succeeded in inducing atrial flutter (AF) by using atrial burst pacing and isoproterenol. Prior to suture line placement, AF was not inducible in all animals, whereas after placement of the atriotomy suture lines, sustainable AF was induced in all dogs; the activation map showed that the AF circuits were dependent on the corridor created between the suture line and the tricuspid annulus [38]. The latter publications by this group using the same model tested two different lines of cryoablation, one from the free wall segment of the TCPC suture line to the tricuspid anulus, in the low lateral right atrium, which was not able to terminate AF, and one from the free wall segment of the TCPC suture line to the tricuspid anulus, incorporating the inferior edge of the atriotomy with much better results (terminating AF in five out of seven cases) [39]. This model hence highlighted the importance of the suture line formed for the intracardiac tunnel in the development of supraventricular tachycardia after the Fontan procedure. However, it still remained an acute model without the effect of atrial dilatation or tissue remodeling [38,39]. In 2019, Wu et al. explored the electrophysiological and histological changes in a canine model of atriopulmonary Fontan connection [40]. The authors observed spontaneous non-sustainable AF in five out of seven subjects at one-week post-procedure. Electrophysiological studies performed at one week showed a shortening of the atrial refractory period and reduced action potential duration due to changes in ionic channel expression, thereby creating a substrate for atrial arrhythmias; the histological study showed fibrotic remodeling after only one week [40]. The same team studied calcium homeostasis in atrial cells 14 days after the Fontan procedure in the same canine atriopulmonary connection model [41]. Their findings highlighted modifications in calcium handling potentially triggering atrial arrhythmias.

While all the above models had concordant results, the technique used in univentricular heart diseases since its first publication in 1996 has nonetheless dramatically evolved from an intracardial tunnel to extracardiac TCPC, which has completely changed the constraints on atrial electrophysiological remodeling.

Indeed, one of the pitfalls to keep in mind is that these animals have a normal cardiac anatomy, and the anatomical and physiological influences of complex congenital cardiac malformations and the hemodynamic alterations after TCPC procedures may have influenced the electrophysiological results. The electrophysiological properties of various species have been extensively studied, for which certain particularities need to be considered. The distribution of the Purkinje fibers in swine extends over nearly the entire transmural distance from the endocardium to epicardium, and, consequently, ventricular activation differs markedly from that in humans. However, Purkinje fibers and cardiac activation sequences in dogs are quite similar to in humans, making canine models reliable for modeling atrial fibrillation or cardiotomy-related scars [8].

### 4.4. Single-Ventricle Failure

One of the hypotheses of acute ventricular dysfunction in the Fontan procedure is a lack of coronary perfusion secondary to an elevated coronary sinus pressure related to high right atrial pressure. The first experimental study addressing this issue was published in 1983 by Ilbawi et al. with a simple montage connecting a blood reservoir to the coronary sinus: by varying the height of the reservoir, they were able to modulate coronary sinus pressure in a canine model of right atrium pulmonary artery anastomosis. The authors observed a significant drop in coronary blood flow and left ventricular ejection fraction when the coronary sinus pressure was above 15 mmHg [42]. In 2002, Szabo et al. confirmed these results with a canine TCPC model and highlighted the importance of the balance between coronary artery and coronary sinus perfusion pressures for single-ventricular function [43].

### 4.5. Liver Disease

In 1994, Higashiyama et al. designed a study to understand Fontan-circulation-related hepatic failure, a common cause of failing Fontan [44]. They used a canine model whereby the thoracic IVC was clamped above the liver and an extracorporeal bypass was inserted to control the level of congestion. The authors determined that a pressure into the IVC of above 27 mmHg changed the energetics of liver hemodynamics. This study allowed for an overview of the acute hepatic aspect of venous congestion observed in the Fontan circulation.

### 4.6. Arteriovenous Fistulae

In 2002, Malhotra et al. studied the development of arteriovenous collaterals in partial cavopulmonary connections (SVC to RPA) through comparison with pulmonary artery banding, in a lamb model [45]. Their findings confirmed the development of an arteriovenous shunt in all animals with cavopulmonary connection, as determined via contrast echocardiography eight weeks after surgery. They also showed an upregulation of gene expression related to endothelial activation, enhanced pulmonary angiogenic signaling, and hypoxemia-inducible factor 1α in operated animals [45].

In a lamb model of SVC-RPA anastomosis, McMullan et al. performed an anatomical study of the arteriovenous fistulae by applying contrast transthoracic echocardiography between 1 and 27 weeks after surgery [46]. The authors observed the same results with intrapulmonary shunts after the fifth week for the entire cavopulmonary anastomosis group. Vascular corrosion casting after sacrifice was performed to anatomically analyze these arteriovenous shunts and revealed arteriovenous shunts only in the lung on the side of the anastomosis bypass shunt [46].

### 4.7. Failing Fontan

**Figure 6:**
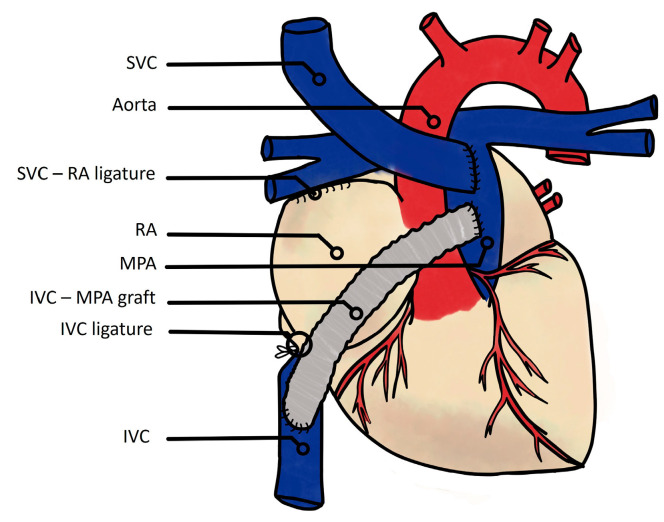
Van Puyvelde’s model of the Fontan circulation, a unique chronic model. Derivation of the SVC to the MPA, and the IVC to the MPA with prosthetic conduct; both vena cava ostias are ligatured [47]. SVC: superior vena cava; RA: right atrium; MPA: main pulmonary artery; IVC: inferior vena cava.

The only published animal model of a chronic TCPC, developed by Van Puyvelde et al. in 2019, uses an extracardiac Y-graft TCPC in a sheep model (Figure 6) [47]. The authors proceeded with an exercise test before and three weeks after the TCPC procedure and used a cardiac MRI to study ventricular function. Ultimately, two-thirds of the animals did not survive the 21st week. Test exercise revealed a significant drop in effort tolerance, although, surprisingly, systemic cardiac output increased, as did left ventricular stroke volume. Although mortality was high in this study, it is the first to report successful animal survival up to 21 weeks after a TCPC procedure.

## 5. Animal Models Assessing Mechanical Circulatory Support in the Fontan Circulation

The first team to address circulatory support in the Fontan circulation was that of Brutel de la Rivière et al. in 1983. The authors hypothesized that hemodynamics would improve if a pulsatile flow was maintained in the pulmonary circulation [48]. They developed a canine model of the Fontan circulation by excluding the right ventricle via suturing of the tricuspid valve, and subsequently added a valved conduit between the RA and the MPA, were the pulsatility assisting device was placed. The results showed a significant increase in cardiac output as well as a pulmonary vascular resistance decrease. Of note, six of the eight experimental animals had complications with an atrioventricular block at the time of tricuspid valve closing [48].

**Figure 7:**
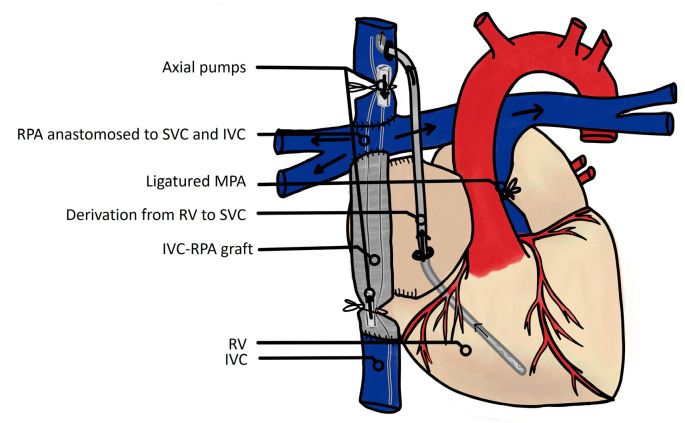
Rodefeld’s model of the Fontan circulation mechanical circulatory support, with two axial pumps draining venous blood from caval veins to pulmonary arteries and a derivation of residual venous blood of the ventricle to the vena cava [49]. SVC: superior vena cava; RPA; right pulmonary artery; RV: right ventricle; MPA: main pulmonary artery; IVC: inferior vena cava.

More than 10 years later, Rodefeld et al. published several articles on this topic [49,50]. Their investigations began in 2003 by assessing the feasibility of circulatory assistance of the Fontan procedure. In a Fontan model consisting of an extracardiac tube connecting the SVC and IVC, which were anastomosed to the RPA, two axial flow pumps were placed within the jugular and femoral veins to provide mechanical circulatory support (Figure 7).

The authors first demonstrated the feasibility of circulatory assistance on 50 kg yearling sheep [49], and similarly the following year on neonatal lambs [50]. In this latter instance, due to the size of the animals, a centrifugal pump was used, allowing perfect hemodynamic stability according to the authors. The next step was to evaluate the consequence of changing the pulmonary vascular resistance parameters with a setting favoring pulmonary vascular constriction or dilatation, which also showed remarkable hemodynamic stability with the circulatory support. Finally, the authors added pulsatility to the mechanical circulatory support and compared the latter to the lambs with continuous assistance flow, and highlighted the absence of significant hemodynamic difference [49,50].

Several authors subsequently studied the use of a left-ventricle assistance device (LVAD) such as the Heartmate II (Thoratec, Bedford, MA, USA), an intrathoracic axial pump usually indicated in left-sided cardiac failure. Riemer et al. assessed the feasibility of the Heartmate II in Fontan circulation consisting of a Y-shaped polytetrafluoroethylene graft between the SVC, IVC, and MPA, with good acute hemodynamic results [51].

Tsuda et al. observed a reduction in lactatemia and an improvement in renal and hepatic function up to 4 days following implantation of a Heartmate II device in sheep [52]. The same team succeeded in keeping the sheep alive up to 18 days post-surgery with a satisfactory effect of the HeartMate II device. Nevertheless, this longer duration of circulatory support was associated with thrombotic and/or hemorrhage complications [53].

Similarly to the Heartmate II, but with the advantage of being smaller and more suitable for children, Derk et al. [54] in sheep and Gondolfo et al. [55] in pigs, respectively, implanted the Jarvik 2000 and the Jarvik Child ventricular assist devices within the Fontan circulation (Y-PTEF montage), with the same technical success and effectiveness albeit with similar thrombotic issues as reported with the Heartmate II.

Other authors focused on the adaptation of the Impella circulatory device to the Fontan circulation, and succeeded in a pig model by using a Y-shaped montage (conduit connecting the SVC and the IVC to the MPA) [56].

In addition, Wang et al. studied the feasibility and results of circulatory assistance by testing the Avalon Elite double-lumen canula, paired with a centrifugal pump in a Fontan circulation sheep model. Input holes were placed percutaneously into the SVC and IVC, and the output hole was placed in front of the pulmonary artery, with satisfactory results with regard to hemodynamic parameters [57].

Lastly, Sinha et al. reported the use of ECMO to assist the failing single ventricle in a more familiar manner (connected between the IVC and the aorta) but still with a model of the Fontan circulation, with good results for the output and improvement of pulmonary arterial pressures [58].

## 6. Discussion and Perspectives

This review is, to the best of our knowledge, the first to present a comprehensive overview of experimental animal models of the Fontan circulation, according to their various objectives. This is an important contribution because designing a reliable experimental protocol requires (1) the ability to identify the most appropriate animal model and surgical set-up to successfully complete the study and validate a scientific hypothesis, and (2) the ability to balance, as much as possible, the conflict between financial and methodological constraints [8]. Notwithstanding, it should be taken into consideration that animal models can closely reproduce both the electrophysiological and mechanical abnormalities found in clinical practice, although they are not a perfect surrogate for the human being. The main limitations in experimental studies with animal models are linked to the differences between human and experimentally induced disease, both in terms of genetic regulatory mechanisms as well as factors that influence cardiovascular function [9].

All of the aforementioned animal studies described in the present review carry limitations, which must invariably be considered during research. The cited experimental models that we examined were typically normal, free of congenital heart disease, tricuspid atresia, or univentricular heart condition. Moreover, the infra-diaphragmatic venous return after the Fontan procedure is highly influenced by activities of normal daily life, including respiration and posture [59,60,61]. Lastly, several studies described in this manuscript included a small number of animals (≤5) due to inherent logistical, technical, or financial factors [18,29,53,54,58,62]. The conclusions drawn from these papers should, therefore, be framed by this limitation.

There is a growing population of failing Fontan patients that will require innovative and creative technological advancements that are capable of supporting their Fontan circulation. In this regard, a chronic failing Fontan experimental model will allow for studying the possible histological changes to the heart, lungs, liver, and other organs after the procedure.

At the same time, it is extremely difficult to establish an acute Fontan circulation in normal hearts, with only one study describing a reliable chronic experimental model [47]. The reason may lie in the fact that, in the clinical setting, the heart is already univentricular prior to the Fontan procedure and may accordingly be adapted to this specific circulatory situation. Approaches facilitating a stepwise adaptation of the cardiovascular system to the altered hemodynamic condition may represent a promising alternative. A combined surgical and transcatheter preconditioning procedure to create a chronic Fontan animal model may have the potential of overcoming the limitations of a solely surgical TCPC completion performed in a single step.

One of the main contributors to the late failure of the Fontan circulation is the increase in PVR. The long-term consequences of PVR are fast becoming a clinical concern as these patients have a longer survival. As the exploration of PVR modulators continues, the need for more trials is paramount. Given the maintained response to nitroprusside in non-pulsatile or low-pulsatile circulations, as demonstrated by Henaine et al., the use of exogenous NO agents may potentially impede the long-term increase in PVR in Fontan patients [37]. As stated by the authors, “because nonendothelial-dependent vasorelaxation was maintained, endothelial dysfunction could be counterbalanced. Therefore, nonendothelial-dependent relaxing agents might represent a potential therapy for failing Fontan circulation with an elevated PAP” [37]. In the future, mechanical assist devices that can re-establish a biventricular physiology could play an important role in the management of failing Fontan patients and serve as a bridge-to-recovery, bridge-to-transplant, or destination therapy. Currently, there are no clinically available Fontan assist devices despite an increasing preclinical interest. Until such time, having a reliable chronic failing TCPC animal model will be essential for investigating the efficacy of Fontan assist devices and various mechanical support strategies.

Computational fluid dynamics and in vitro experimental circuits of the Fontan circulation have played a significant role in the investigation of the hemodynamic characteristics of the Fontan procedure and have been applied to the design and integration of the procedure [13,55,62,63,64]. At a time when ethics as well as financial and technological constraints are indubitable features of scientific research, the pursuit of in vitro and in silico research remains essential.

## 7. Conclusions

The establishment of the experimental Fontan procedure, given the physiological anatomy of the heart, is highly challenging and extremely difficult, despite the wide spectrum of studies with highly diverse experimental animal models in the literature. Nonetheless, animal models have allowed for the evolution of the Fontan operation’s design and improved our knowledge of its physiopathology. They also support us to assess future developments that may improve the contribution of animal models to the testing of new therapeutic strategies. In the future, an experimentally validated chronic model will constitute an essential step forward.

## Appendix A

**Table A1:** Summary of the most relevant studies included in this review, according to their aim, type of model used and main results.

Study	Year	Species Model	Number of Animals	Weight (kg)	Type of Fontan Procedure	Purpose	Surgical Technique	Pump Type	Maximal Follow-Up	Main Results
Van Puyvelde J et al. [47]	2019	**Sheep**	15	median 63.4 [IQR 62.8–68.5]	TCPC	Feasibility of chronic model	Surgery: End-to-side anastomosis SVC-PA, ePTFE conduit IVC-PA and IVC-RA junction ligated	-	21 weeks	Feasibility of chronic model (10 deaths)
Gandolfo F et al. [55]	2016	**Sheep**	8	Range: 42–48	TCPC	Feasibility and efficacy of axial pump	Surgery: T-shaped model (Gore-Tex tube)—Axial pump positioned in the latter conduit	Jarvik Child 2000	3 h	The axial flow pump provides normal CO and physiologic stability
Wang D et al. [57]	2014	**Sheep**	6	Range 35–45	TCPC	Feasibility of designed double-lumen cannula with paired umbrellas without alignment requirement	Surgery: ECC Gore-Tex 18 mm	Avalon Elite™ double lumen cannula	-	Feasibility of designed double-lumen cannula with paired umbrellas without alignment requirement: easy to insert and remove
Boudjemline Y et al. [21]	2014	**Sheep**	20 (9 + 11)	Mean 20 ± 2.5	TCPC	Feasibility of acute hybrid model	Hybrid: Anastomosis IVC-Gore-Tex conduit and connection of SVC with RA. Group 1 = Gore-Tex conduit occluded at both ends by a PTFE membrane—Group 2 = upper segment of Gore-Tex tube opened and connected to RA. Fontan completion 1–3 months following surgery	-	3 months	Group 1: failure of completion in all models (occlusion of the pathway)—Group 2: 9 successful completions
Boudjemline Y et al. [20]	2013	**Sheep**	16 (10 + 6)	Mean 55 ± 7	**Cavocaval connection**	Feasibility of 2 various hybrid models	Hybrid: Group 1 = ICC, Group 2 = ECC (20 mm Gore-Tex)	-	Median 4 months (range 0–8 months)	Both techniques were equally successful with no failures or short-term complications
Gerelli S et al. [22]	2013	**Sheep**	10	-	**Cavocaval connection**	Feasibility	Hybrid: SVC–RA junction closed using a PTFE membrane. A valveless Gore-Tex conduit connecting the terminal portion of the SVC to the RA (bypass the PTFE occlusion and allow normal venous drainage through the RA). Radio-opaque nitinol rings (around IVC)	-	median, 5 months	Feasibility of chronic ovine model of cavocaval connection
Boni L et al. [53]	2012	**Sheep**	5	Range: 46–63	ICPC	Efficiency of longer cavopulmonary support	Surgery: IVC-MPA connection by PTFE	Thoratec Heartmate II	18 days	Efficiency of longer cavopulmonary support (death at 2, 4, 8, 10 and 18 postoperative day)
Metton O et al. [19]	2010	**Sheep**	16	Mean 53 ± 6	TCPC	Feasibility of hybrid model	Hybrid: Connection SVC-PA using a Gore-Tex conduit. Connection interrupted between SVC and RA (vascular stent occluded in the middle by a PTFE membrane), transcatheter completion (perforating the membrane of the occluded stent and placing covered stents from IVC to the SVC stent)	-	-	Feasibility of the hybrid model
Tsuda S et al. [52]	2009	**Sheep**	10	Range: 47–57	TCPC and ICPC	Feasibility mid-term survival with mechanical circulatory support	Surgery: ICPC (Dacron graft for anastomosis main PA-pump outflow conduit and PTFE for anastomosis IVC-pump inflow conduit), TCPC (Additional SVC-main PA anastomosis by PTFE graft)	Thoratec HeartMate II	4 days	Feasibility mid-term survival with MCS (7 deaths)
Riemer RK et al. [51]	2005	**Sheep**	8	Range: 42–48	TCPC	Efficiency of acute model and feasibility of mechanical circulatory support	Surgery: SCPC with PTFE, ligating SVC-RA and IVC-RA junctions, connection IVC-main PA with PTFE. Axial flow pump between IVC inflow cannula and the main PA	Thoratec HeartMate II	1 h	Axial flow pump support can prevent the substantial decrease in aortic flow and pressure
Rodefeld MD et al. [50]	2004	**Sheep (lambs)**	13	Mean 5.6 ± 1.5	TCPC	Efficiency of pump assist	Surgery: bicaval venous-to-main pulmonary artery cannulation, cannula RV-systemic venous circulation	Miniature centrifugal pump (Paraflow; A-Med Systems Inc.)	8 h	CI, SAP, LAP, lactate stable;mPAP and PVR increased
Rodefeld MD et al. [49]	2003	**Sheep**	13	Range: 33–78.6	TCPC	Efficiency of mechanical circulatory support	Surgery: Direct anastomosis SVC-RPA and PTFE from IVC to RPA. Axial flow pumps positioned within both vena cavae. Proximal main PA clamped. RV decompression by a cannula (RV-Systemic venous circulation).	2 Hemopumps (HP-24, 24F; Medtronic Inc., Minneapolis, MN, USA)	6 h	Hemodynamic stability without the need for volume loading, inotropic support, or pulmonary vasodilator therapy in all animals. CO, PVR, PAP, CVP and arterial pCO2 and pO2 values 6 h after intervention were similar to baseline values.
Malhotra SP et al. [34]	2002	**Sheep (lambs)**	6	-	Unilateral SCPC	Determine which genes are upregulated independent of reduced pulmonary blood flow and role of hypoxia-inducible factor1α in the development of PAVS	Surgery: SVC-RPA connection, proximal RPA ligation	-	8 weeks	Increased angiogenic gene expression and expression of endothelial stress-related genes (including HIF-1α)
Malhotra SP et al. [35]	2001	**Sheep (lambs)**	12	-	Unilateral SCPC	Assess regulation of pulmonary vascular tone	Surgery: SVC-RPA connection, proximal RPA ligation	-	1, 2, 5, and 15 weeks	Early reversible reduction in activity and expression of angiotensin-converting enzyme
Klima U et al. [17]	2000	**sheep**	10	Mean 35 ± 6	TCPC	Feasibility of hybrid model	Hybrid: SVC-RPA anastomosis by a PTFE and interventional catheter completion (stent graft from IVC to SVC-RPA anastomosis)	-	-	Feasibility of the hybrid model
Wei X et al. [56]	2014	**Pig**	6	Mean 8.8 ± 0.9	TCPC	Feasibility and efficiency of a miniature axial flow pump	Surgery: SVC and IVC to PA connection with a 12 mm Dacron conduit with two branches. Impella pump inserted into the conduit through an additional PTFE graft in 5 animals	Impella pump	6 h	Feasibility and efficiency of a miniature axial flow pump: 4 pump-supported animals remained hemodynamically stable for 6 h (1 death)
Sinha P et al. [58]	2014	**Pig**	5	25	TCPC, ICPC and SCPC	Efficiency of mechanical circulatory support	Surgery: Creation of an atrial septal communications, CPC with a PTFE conduits in a Y configuration and snare on the main PA	Continuous flow VAD	-	SCPC allowed optimal increase in CI and oxygen delivery
Derk G et al. [54]	2014	**Pig**	4	Range: 44–61	TCPC	Efficiency of axial flow ventricular assist devices	Surgery: Synthetic grafts from IVC and SVC to main PA-VAD implanted within the common Fontan graft to provide a pulmonary pump	Jarvik 2000	-	Restoration of normal hemodynamics and cardiac output
Vikholm P et al. [65]	2014	**Pig**	11	36.6 ± 2.9	SCPC	Evaluation of SCPC in RV failure	Surgery: PA banding and then vascular clamp on the SVC proximal to the shunt, diverting the venous return from the upper body directly to the PA.	-	2 h	Reduced RA pressure and venous congestion
Henaine R et al. [37]	2013	**Pig**	20	Mean 20 ± 2	Unilateral SCPC	Effect of pulsatility on pulmonary vasculature	Surgery: SVC-RPA connection-proximal RPA ligation or banded proximal RPA	-	3 months	Altered endothelial-dependent vasorelaxation response. Micropulsatility limited the effects of pulsatility loss
Kanakis MA et al. [16]	2011	**Pig**	8	43 ± 3.8	TCPC	Feasibility and efficiency of acute model	Surgery: Y-shaped conduit connecting and draining IVC and SVC to the pulmonary trunk, coronary venous blood collected and directed into the pulmonary flow of the graft by use of a small roller pump	-	1 h	Feasibility of the model—Increase in PVR and CVP—Decrease in CO and PAP
Corno AF et al. [62]	2010	**Pig**	4	Range: 57–68	ICPC	Efficiency of the model on pulmonary bloodflow	Surgery: connection by Contegra conduit extended with PTFE (Gore-Tex) tubular prosthesis between IVC and PA wrapped with latissimus dorsi	Latissimus dorsi muscle wrapped around ICPC	-	2% increase in blood flow
Konstantinov IE et al. [18]	2005	**Pig**	4	Range 15–30	TCPC	Feasibility of hybrid model	Hybrid: SVC anastomosed to RPA, 2 stents placed around atriocaval junctions and interventional catheter completion (covered stent graft expanded against the superior and inferior stents)	-	-	Feasibility of the hybrid model
Macé L et al. [30]	2000	**Pig**	8	Mean 30.2 ± 1.4	TCPC	Hemodynamic evaluation of biventricular and univentricular circulation	Surgery: Y-shaped conduit-proximal part of the divided pulmonary trunk anastomosed to the LA appendage to divert the CS flow into the LA	-	-	PVR induced a proportional decrease in venous return conductance and volume loading, while increasing mean circulatory filling pressure allowed the venous return conductance gradient to increase and maintain systemic blood flow at a biventricular level
Randsbaek F et al. [66]	1996	**Pig**	6	Range: 3.5–6.0	Univentricular heart without CPC	Effect of ventilatory mechanics on Qp/Qs ratio	Surgery: atrial septostomy, tricuspid valve rendered incompetent, occluding RV outflow, and anastomosis between MPA and brachiocephalic artery	-		
Zhou W et al. [41]	2018	**Dog**	14	Range 15–20	TCPC	Evaluation of atrial cardiomyocyte calcium handling remodeling	Surgery: Atriopulmonary anastomosis by a Gore-Tex tube	-	2 weeks	Atrial cardiomyocyte Ca^2+^ handling abnormalities that produced arrhythmogenic-triggered activity and increased vulnerability to atrial tachycardia (4 deaths)
Zongtao Y et al. [36]	2010	**Dog**	20	Range: 15–20	Unilateral SCPC	Evaluation of microvascular bed of the pulmonary circulation under nonpulsatile flow perfusion	Surgery: SVC-RPA anastomosis, proximal end RPA ligated	-	6 months	eNOS synthesis decreased in endothelial cells—Apoptosis of smooth muscle cells increased and led to arterial venous conversion and pulmonary vessel remodeling
Szabo’ G et al. [33]	2003	**Dog**	12	Mean 22.3 ± 5.2	TCPC	Evaluation of cardiac performance	Surgery: Y-shaped conduit—Cannula in RV to collect coronary venous blood, which was directed into the external jugular vein with a small roller pump	-	-	Contractility-afterload mismatch
Szabo’ G et al. [43]	2002	**Dog**	6	-	TCPC (bypass extracorporeal circuit with height adjustable reservoir)	Evaluate relationship between coronary perfusion pressure and myocardial contractility	Surgery: Bypass circuit (PA proximal cannulation and both caval veins cannulation + coronary arteries cannulated and perfused with a pressure controlled roller pump)	-	-	Both coronary arterial and venous pressure affected myocardial contractility
Voss B et al. [26]	2002	**Dog**	19	Range: 18.4–26	TCPC	Efficiency of RA cardiomyoplasty	Surgery: RA-PA trunk connection by a valveless conduit, tricuspid valve closed with a patch-mechanical valve (Medtronic Hall 16 mm) implanted at the inferior cavo-atrial junction or not	Latissimus dorsi muscle over RA	-	Feasibility of a ‘ventricularization’ of the RA. Hemodynamic benefit achieved only when a valve was implanted in the inferior cavo-atrial junction
Morita K et al. [25]	2001	**Dog**	8	Range: 9–15	TCPC	Efficiency of a dynamic cardiomyoplasty	Surgery: RV free wall totally excised and RV cavity obliterated by plication sutures after closure of tricuspid valve and RV outflow tract, aortic homograft and xenopericardial pouch as neo-RV chamber between RA and PA trunk	left latissimus dorsi	4 h	Marked augmentation of right heart performance, restoring almost normal pulmonary circulation
Tanoue Y et al. [14]	2001	**Dog**	24	Mean 16.8 ± 2.7	TCPC (bypass extracorporeal circuit)	Validate the accuracy of the approximation of end-systolic elastance (contractility index) and effective arterial elastance (afterload index).	Surgery: Tapes around SVC and IVC snared to direct the systemic venous blood return into a reservoir and then pumped back to the main PA	Centrifugal pump	-	High correlations
Rodefeld MD et al. [38]	1996	**Dog**	17	Range: 25–30	No CPC (lateral tunnel suture)	Explore lateral tunnel suture line and characterize pathways of resultant arrhythmias	Surgery: Total cavopulmonary connection suture line was placed through a standard right atriotomy, simulating construction of the lateral tunnel.	-	-	Lateral tunnel suture line sufficient to create an anatomic substrate for the development and maintenance of atrial flutter (around venae cavae)
Rodefeld MD et al. [39]	1996	**Dog**	7	Range: 25–30	Non CPC (suture line of lateral tunnel)	Efficiency of a cryolesion for terminate atrial flutter	Surgery: Total cavopulmonary connection suture line was placed through a standard right atriotomy, simulating construction of the lateral tunnel.	-	-	Efficiency of linear cryothermal lesion placed between the free wall aspect of the TCPC suture line and the tricuspid anulus
Macé L et al. [29]	1995	**Dog**	5	Mean 22.4 ± 1.5	SCPC, ICPC and TCPC	Hemodynamics evaluation	Surgery: Y-shaped conduit + total right heart exclusion (ASD, posterolateral flap of the RA wall sutured to the atrial septum, anterior wall of the RA sutured over the posterolateral flap)	-	2 h	Increasing degrees of right heart bypass associated with significant decrease in ventricular performance
Haneda K et al. [28]	1993	**Dog**	7	Range: 7.8–14.8	TCPC	Hemodynamics evaluation	Surgery: Occlusion TV, RA-PA connection (Gore-Tex graft)	-	-	Decrease: PAs and CO; Increase: PVR
Kaku K et al. [27]	1990	**Dog**	9	Range: 7.3–10.3	TCPC	Evaluation of hemodynamic responses with vasoactive drugs	Surgery: Artificial graft between SVC and IVC, anastomosis of SVC to RPA, IVC and pulmonary trunk snared, coronary venous return shunted from RV outflow tract to LA	-	-	Isoproterenol and phentolamine: increased CO, decreased PVR and SVRNorepinephrine: detrimental, increased PVR and SVR
Bridges CR Jr et al. [67]	1989	**Dog**	7	Range: 12–20	TCPC and ICPC	Efficacy of a ventricle constructed from skeletal muscle	Surgery: Connection IVC and SVC to the skeletal muscle ventricle, outflow from the SMV directed into PA (pacing system stimulated SMV to contract)	SMV (latissimus dorsi)	8 h	Efficacy of the SMV
Brutel de la Riviere A et al. [48]	1983	**Dog**	8	29.8 ± 1.6	TCPC	Efficiency of counterpulsation	Surgery: RA-PA connection with valved conduit Hancock, tricuspid valve sutured	Pulsatile assist device connected to a System 82 PAD console, Datascope Corp., Paramus, N. J.	-	Decreased PVR
Shemin RJ et al. [13]	1979	**Dog**	20	Range: 22–28	TCPC	Efficiency of RA-PA conduits	Surgery: Patch closure of 90% of the tricuspid valve orifice, 6 months later: RA-PA shunting with a conduit with paralled limbs, one containing a prosthetic valve, main PA ligated	-	-	RAP = most important factor influencing conduit flowAbsence of a valve, ventricular rhythm and tachycardia did not alter flow
